# Impact of Methanol Concentration on Properties of Ultra-Nanocrystalline Diamond Films Grown by Hot-Filament Chemical Vapour Deposition

**DOI:** 10.3390/ma15010005

**Published:** 2021-12-21

**Authors:** Lidia Mosińska, Robert Szczęsny, Marek Trzcinski, Mieczysław Karol Naparty

**Affiliations:** 1 Institute of Physics, Kazimierz Wielki University, Powstańców Wielkopolskich 2, 85-090 Bydgoszcz, Poland; 2 Faculty of Chemistry, Nicolaus Copernicus University in Torun, Gagarina 7, 87-100 Torun, Poland; Robert.Szczesny@umk.pl; 3 Institute of Mathematics and Physics, Bydgoszcz University of Science and Technology, Al. S. Kaliskiego 7, 85-796 Bydgoszcz, Poland; marekt@pbs.edu.pl (M.T.); naparty@pbs.edu.pl (M.K.N.)

**Keywords:** ultra-nanocrystalline diamond, hot filament chemical vapour deposition, nanoparticles, trans-polyacetylene

## Abstract

Diamond is a very interesting material with a wide range of properties, making it highly applicable, for example, in power electronics, chemo- and biosensors, tools’ coatings, and heaters. Due to the high demand for this innovative material based on the properties it is already expected to have, it is important to obtain homogeneous diamond layers for specific applications. Doping is often chosen to modify the properties of layers. However, there is an alternative way to achieve this goal and it is shown in this publication. The presented research results reveal that the change in methanol content during the Hot Filament Chemical Vapour Deposition (HF CVD) process is a sufficient factor to tune the properties of deposited layers. This was confirmed by analysing the properties of the obtained layers, which were determined using Raman spectroscopy, scanning electron microscopy (SEM), X-ray diffraction (XRD), and an atomic force microscope (AFM), and the results were correlated with those of X-ray photoelectron spectroscopy (XPS). The results showed that the increasing of the concentration of methanol resulted in a slight decrease in the sp^3^ phase content. At the same time, the concentration of the -H, -OH, and =O groups increased with the increasing of the methanol concentration. This affirmed that by changing the content of methanol, it is possible to obtain layers with different properties.

## 1. Introduction

Today, the search for new materials with specific properties is still ongoing. The materials can be divided into two main groups: organic and inorganic. Each of these groups is characterised by specific properties and can be used to build specific devices [1,2]. In recent years, interest in the preparation and investigation of properties of thin film structures has significantly grown. This has allowed the creation of new devices such as sensors, detectors, optoelectronic elements, and electrochemical electrodes [3,4,5,6,7,8]. The possibility of surface functionalisation has enabled the creation of selective sensors operating in specific conditions [9,10].

Currently, there are many materials, which are used as electrode materials for chemical sensors. Diamond films are, among other materials, studied for this purpose. The challenge for thin diamond layers is to ensure both high sensitivity and selectivity as well as high electrochemical stability and resistance to aggressive environments [11]. CVD diamond layers also find new applications as protective coatings in micro electrochemical devices, supercapacitors, and fuel cells [12]. Ultra-nanocrystalline diamond (UNCD) layers are considered as a prospective electrode material due to its wide band gap, high breakdown voltage, and small dielectric constant [13,14,15,16]. It is necessary to tune the mechanical, electrical, and chemical properties to specific applications. The most common strategy for modifying CVD diamond layers is doping with boron and nitrogen [17,18], which results in a Fermi level shift and changes in the band gap. A wide range of physical or mechanical properties of CVD diamonds depends on the ratio of the *sp*^2^ and *sp*^3^ phase [19,20,21,22].

Recently, several efforts have been made to reduce diamond grain sizes, as smaller grain sizes give better electrochemical properties [23]. This paper describes the synthesis of UNCD films using the hot filament chemical vapour deposition (HF CVD) technique. Methanol is used as a carbon precursor. We show that the UNCD grains grown during the HF CVD process can be easily controlled by changing the methanol concentration in the reaction gas mixture (H_2_/CH_4_OH), which, in this study, ranged between 4 and 7 vol%.

## 2. Materials and Methods

### 2.1. HF-CVD Growth

The diamond films were synthesized in a home-made HF CVD reactor. As a filament wire with a cross section of 0.5 mm in a form of spring, with a coil diameter of 5 mm, tungsten was used. Quartz plate (100) was used as a substrate, with dimensions of 5 × 5 mm^2^. Before being placed in the reaction chamber, quartz substrate was subjected to an ultrasonic bath in chloroform for 4 min, in a suspension of diamond powder in methanol for 6 min, and finally in methanol for 6 min. The time deposition of each sample was 6 h. The mixture of methanol vapour and hydrogen (CH_3_OH/H_2_) was used as a working gas. CVD synthesis was carried out at total pressure of 50 mbar, a substrate temperature of ~1000 K, and a working gas flow rate of 100 sccm. The methanol concentration in working gas was changed between 4 and 7 vol%. Thus, the fine-tuning of the properties of this type of layer by using only one process parameter allowed the fabrication of films with various properties.

### 2.2. Material Characterisation

The surface morphology was investigated by scanning electron microscopy (SEM) using a Quanta 3D FEG (VP mode, SE detector) (FEI, Hillsboro, OR, USA). The particle size was measured using the SEMAFORE program.

Structural information came from X-ray diffraction (XRD) measurements; all materials were also characterised by powder X-ray diffraction (PXD) using Philips XPERT Pro θ-2θ and D8 Bruker diffractometers with CuKα radiation ((PANalytical—Malvern Panalytical Ltd., Malvern, UK).

The surface topography of the UNCD films was examined using the Bruker Innova atomic force microscope (AFM) from Bruker with standard silicon tips for use in tapping mode. The scan size was 5 μm × 5 μm. The mean surface roughness was determined using the NanoScope Analysis software (version 1.40).

The Model 2450 SourceMeter SMU Instrument was used for resistance measurements in 2-point probe configuration. The spacing between electric contacts (tungsten needles) was set to 1 mm.

The Raman spectroscopy measurements were carried out on a Senterra (Bruker Optik GmbH, Ettlingen, Germany) spectrometer using a 532 nm excitation laser. The analysis of the Raman scattering spectra was performed after removing the fluorescent background by means of asymmetric least square smoothing. Characteristic bands were fitted with the Gaussian–Lorentzian cross product function.

X-ray photoelectron spectroscopy (XPS) measurements were performed under ultra-high vacuum (UHV) conditions (base pressure ≤ 2 × 10^−10^ mbar). The spectra were recorded with a VG-Scienta R3000 (Uppsala, Sweden) hemispherical analyser using AlKα (1486.6 eV) as a radiation source. The energy step was set to ΔE = 100 meV. The pass energy of the analyser was set to 50 eV and the spectra were calibrated using the W4f level of 31.6 eV.

## 3. Results

### 3.1. Scanning Electron Microscopy, X-ray Diffraction, and Atomic Force Microscopy

**Figure 1:**
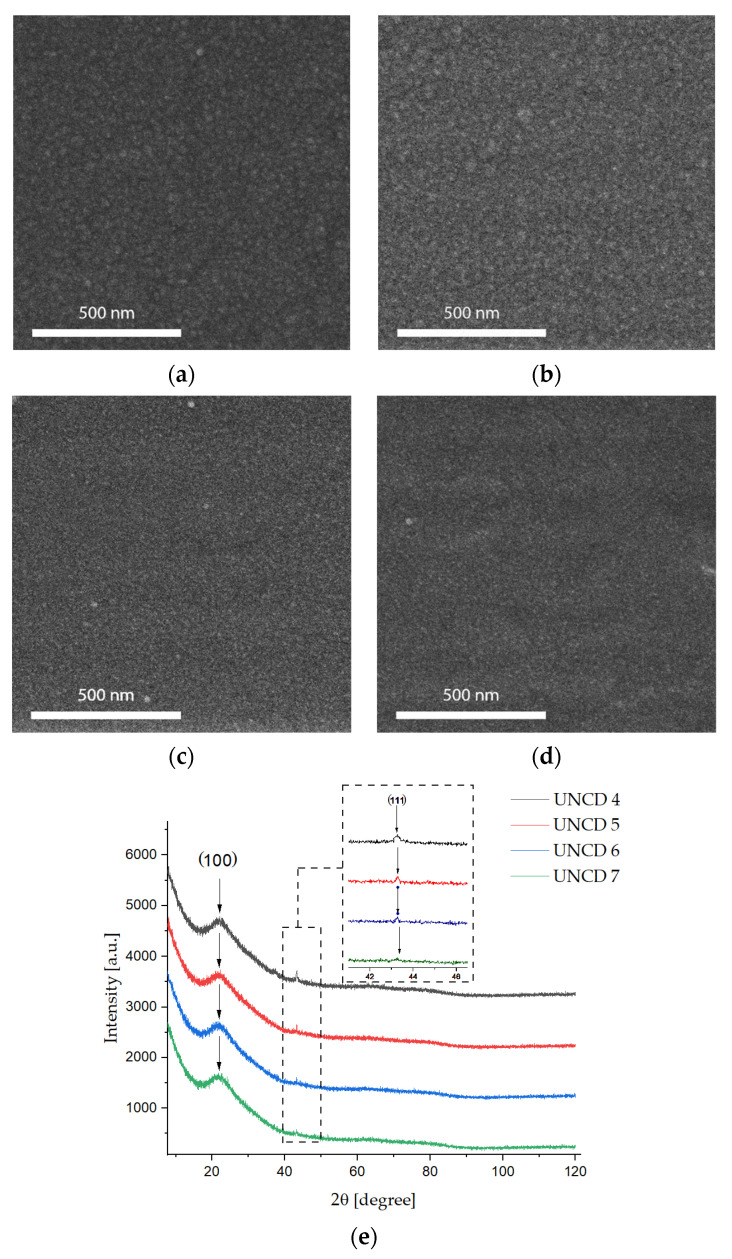
CVD diamond films, with the content of the vapours of methanol in quantities of (**a**) 4% (UNCD-4), (**b**) 5% (UNCD-5), (**c**) 6% (UNCD-6), and (**d**) 7% (UNCD-7), with (**e**) XRD peaks of diamond films.

Figure 1 shows SEM images of the UNCD films grown at different methanol concentrations. The fabricated diamond layers are smooth and the homogenous nanocrystals are clearly visible. The influence of methanol concentration on crystalline size is apparent. The size of UNCD grains, estimated from high-resolution SEM, can be seen to decrease with the increasing of the methanol concentration from 24 ± 2 nm and 13 ± 2 nm (Figure 1a–d).

The sizes of the crystals were determined using XRD, based on Scherrer’s equation. All spectra for the (111) diamond reflexes, with planes at 2θ = 43.5°, were applied and the grain sizes were about 8-3 ± 2 nm, which confirmed the presence of ultranano-sized crystallites [24]. The observed peak from (100), with planes at 2θ = 21.7°, was dedicated to the quartz substrate.

**Figure 2:**
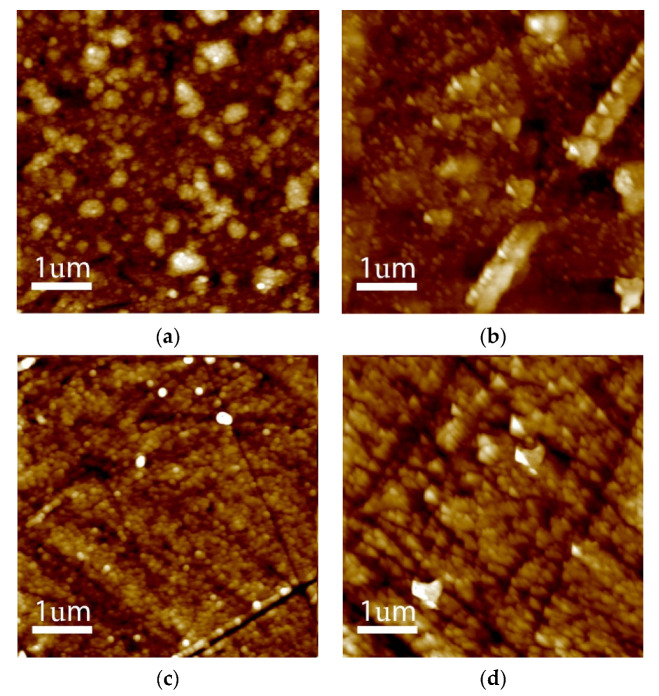
AFM 2D topography of (**a**) 4% (UNCD-4), (**b**) 5% (UNCD-5), (**c**) 6% (UNCD-6), and (**d**) 7% (UNCD-7).

The topographies of deposited layers were also analysed by atomic force microscopy. The mean surface roughness decreased with the increasing of the methanol content. The values of Ra and Rq, for the tested samples (UNCD-4, UNCD-5, UNCD-6, and UNCD-7), were 6.56 nm and 8.42 nm, 2.19 nm and 2.89 nm, 1.17 nm and 1.73 nm, and 1.43 nm and 1.89 nm, respectively. Figure 2 shows the AFM images recorded for the UNCD diamond films, confirming the presence of ultranano-sized diamond crystallites.

**Figure 3:**
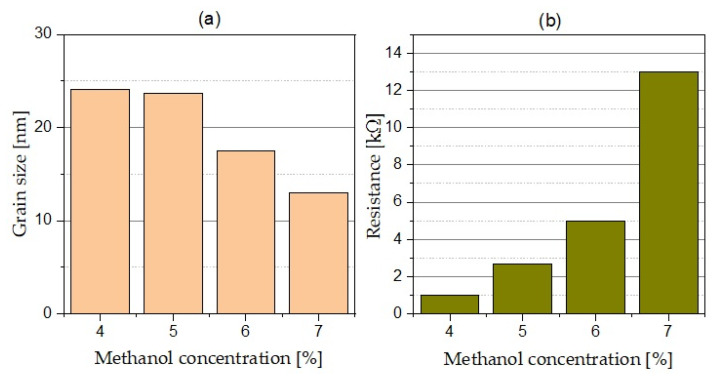
Methanol vapour content for diamond films on the basis of: (**a**) grain size and (**b**) layer resistivity.

Changes in methanol concentration also influenced the surface resistance. Figure 3b compares the resistance measurements performed at the UNCD layers. Slight changes in the concentration of methanol (4–7%) caused a thirteen-fold increase in the surface resistance of the layer. We attribute this behaviour to the decreasing grain size of the diamond, which probably increased the specific surface of the film.

### 3.2. Raman Spectroscopy

**Figure 4:**
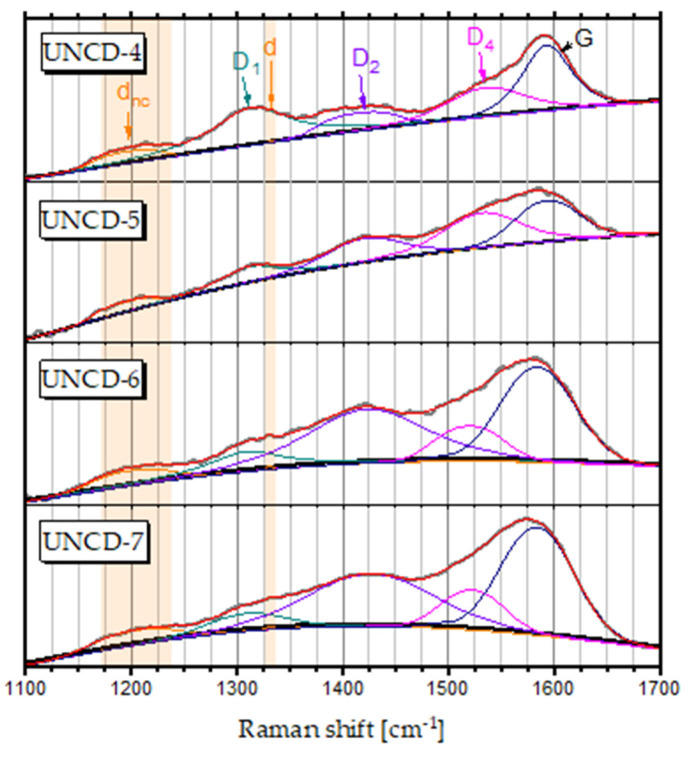
Raman spectra of the UNCD layers grown at methanol concentrations ranging from 4% to 7%. Various components derived from the peak fitting procedure are also depicted using the following colour code: orange for the diamond components (d and d_nc_ components), dark cyan for the first C-H/*sp*^3^ defect-induced graphene component D_1_, violet and magenta for the D_2_ and D_4_ components, and black for graphene’s G-mode.

Raman spectroscopy is the most common method used to characterise the different forms of carbon. Figure 4 shows the Raman spectra of UNCDs grown at different concentrations of the methanol vapours. In addition to a weak sharp diamond peak at 1332 cm^−1^ (denoted as ‘d’), the spectra showed a broad feature at around 1200 cm^−1^ (denoted as ‘d_nc_’) attributed to small diamond clusters [25,26,27]. The position of the d_nc_ peak coincided with the vibrational density of states (VDOS) of diamond at 146 meV (1175 cm^−1^) and 156 meV (1260 cm^−1^) [28,29,30].

The d_nc_ band was Raman inactive in the bulk diamond. However, the selection rules for Raman transitions were broken in UNCD due to the lack of translational symmetry of the crystalline lattice, which gave rise to the observed broad d_nc_ component in the Raman spectra of the tetrahedral carbons [12,31].

The remaining components of the Raman spectra, which are associated with the non-diamond phase, differ essentially from the results already reported for nanocrystalline diamond [13,31,32] These works prove that the *sp^2^* fraction in nanocrystalline diamond is a mixture of amorphous carbon and trans-polyacetylene (t-Pa) macromolecules. The statement was supported by the observed Raman spectra, in which the graphitic D and G bands around 1350 and 1570 cm^−1^ were accompanied with t-Pa bands around 1150 and 1480 cm^−1^. However, this interpretation could not be applied to the Raman spectra of the UNCD grown from methanol. Firstly, no single graphitic D component was found at around 1340–1360 cm^−1^. Secondly, it was impossible to distinguish a band from t-Pa at about 1480 cm^−1^. Instead of those Raman features, we distinguished broad peaks at around 1310 cm^−1^, 1420 cm^−1^, 1525 cm^−1^, and 1590 cm^−1^ that were assigned as D_1_, D_2_, D_4_, and G. We used the assignation proposed by Vecera et al. [33], who performed vibrational Raman response calculations of graphene functionalised with hydrogen. According to Vencera’s calculations, we assigned the D_1_ mode to a fingerprint of the oscillations of the carbon atoms directly surrounding the C-H/*sp*^3^ defect, the D_2_ band that arose due to the oscillations of carbon atoms directly surrounding the C-OH/*sp*^3^ defects, and the next neighbouring atoms that surrounded both the C-H/*sp*^3^ and C-OH/*sp*^3^ defects. The D_4_ mode was assigned to the oscillations of the next neighbouring atoms surrounding the carbon atoms that were functionalised by the H and OH groups. The G-mode arose from the E_2g_ vibrational mode of the graphene lattice.

**Figure 5:**
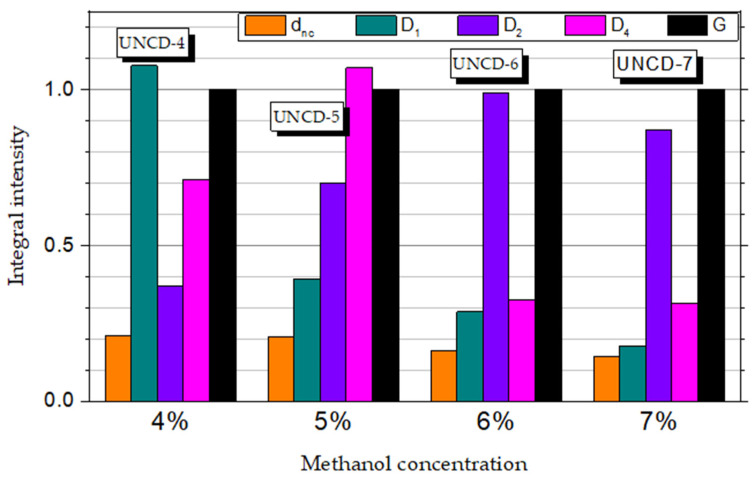
Comparison of integral intensities of Raman features’ components normalised to the intensity of the G band.

Figure 5 presents the development of individual peak intensities as a function of methanol concentration. We observed a slight decrease in intensity of the nanodiamond-related d_nc_ component with the increasing of the methanol concentration. At the same time, a significant decrease in the intensity of the D_1_ line was clearly visible. Thus, we could conclude that the formation of UNCD clusters was coupled with C-H/*sp*^3^ defects. Higher concentrations of methanol in the methanol/H_2_ mixture were not conducive to the process of *sp*^3^ phase formation. On the other hand, higher methanol concentrations resulted in the C-OH/sp^3^ defects that were manifested by the broadening and higher integral intensity of the D_2_ component.

### 3.3. X-ray Photoelectron Spectroscopy

**Figure 6:**
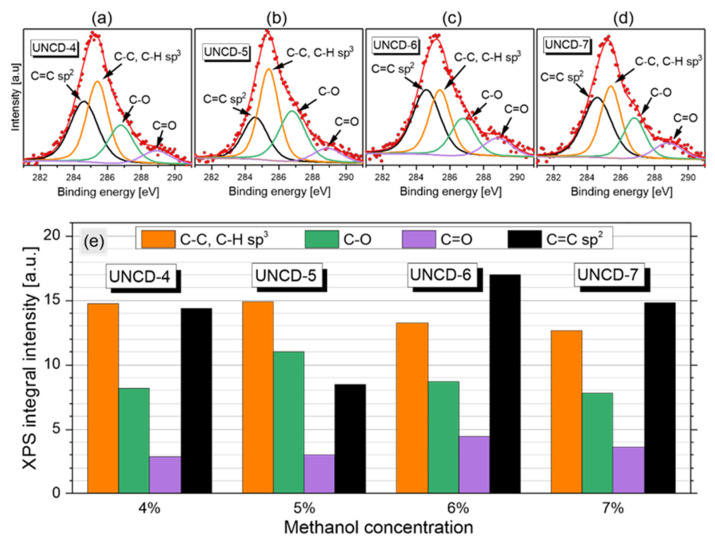
(**a**–**d**) XPS spectra of C 1s peak of UNCD films grown at different methanol concentrations. (**e**) Comparison of the results of C 1s peak deconvolution with quantification of different carbon bonding states.

Figure 6a–d shows XPS spectra of the C 1s peak of the UNCD samples. The results of C 1s peak deconvolution with quantification of different carbon bonding states are compared in Figure 6e. By adjusting the energy allocations, then the Gaussian distribution, four types of bonds of the diamond layers could be distinguished: C=C sp^2^ bonding at 284.5 ± 0.1 eV, C-C, C-H sp^3^ bonding at 285.4 ± 0.1 eV, C-O bonding at 286.8 ± 0.1 eV, and C=O bonding at 288.9 ± 0.1 eV [34].

The XPS data confirmed the conclusions from the analysis of the first-order Raman spectra. Namely, we observed a slight decrease in the intensity of the C 1s peak component attributed to the C-C and C-H bonding of carbon in tetrahedral configuration. Changes in the C=C sp^2^ peak intensity correlated with the G-band intensity in the Raman spectra. On the other hand, no regularity was found in the intensities of the C-O and C=O component of the C 1s peak, which may be due to XPS being a surface technique.

## 4. Conclusions

In conclusion, the presented results show that UNCD properties can be modified by changing the methanol concentration in working gas during the CVD growth process. A slight change in the methanol concentration from 4 to 7% resulted in a reduction in the UNCD grains. At the same time, the resistivity of the films significantly increased, by more than one order of magnitude, which was attributed to the increase in the surface area of the diamond grains. The layers will ultimately be used as a transducer using an electrolyte (as an active layer in the sensor to determine the condition of drinking water), and thus, the minimum amount of admixtures is recommended. The admixtures additionally broke the crystal structure. By changing the methanol content, we changed the properties of the layer, which was our goal.

Broad peaks at around 1310 cm^−1^, 1420 cm^−1^, 1525 cm^−1^, and 1590 cm^−1^ that were marked as D_1_, D_2_, D_4_ and G were visible in the Raman spectra. The XPS results showed four types of bonds of the diamond layers: C=C sp^2^ bonding at 284.5 ± 0.1 eV, C-C and C-H sp^3^ bonding at 285.4 ± 0.1 eV, C-O bonding at 286.8 ± 0.1 eV, and C=O bonding at 288.9 ± 0.1 eV. There was a visible relationship between the results of the Raman and XPS spectroscopy. Changes in the C=C sp^2^ peak intensity correlated with the G-band intensity in the Raman spectra.

The Raman and XPS results showed changes in the structure of UCNDs. Namely, the increasing of the concentration of methanol resulted in a slight decreasing of the sp^3^ phase content. At the same time, the concentration of the -H, -OH, and =O groups increased with the increasing of the methanol concentration.

The presented results confirm that doping is not needed to change the properties of diamond layers. The appropriate selection of process parameters can lead to the modification of the sp^3^/sp^2^ proportion, which makes it possible to obtain layers with the required properties, and this, has very wide applicability.

## Data Availability

Not applicable.
